# Genetic Diversity and Evolution of Porcine Rotavirus Species A in Guangxi Province, Southern China, Between 2022 and 2025

**DOI:** 10.3390/ani16152292

**Published:** 2026-07-23

**Authors:** Yuwen Shi, Junxian He, Kaichuang Shi, Feng Long, Shuping Feng, Yanwen Yin, Wenjun Lu, Sujie Qu, Xingjv Song

**Affiliations:** 1 College of Animal Science and Technology, Guangxi University, Nanning 530005, China; shiyuwen2@126.com (Y.S.); ces5734@yeah.net (J.H.); songxingju@gxu.edu.cn (X.S.); 2 Guangxi Center for Animal Disease Control and Prevention, Nanning 530001, China; fsp166@163.com (S.F.); yanwen0349@126.com (Y.Y.); nnlwj@126.com (W.L.); mingdao120@126.com (S.Q.)

**Keywords:** porcine rotavirus (PoRV), porcine rotavirus species A (PoRVA), phylogenetic analysis, genetic evolution, recombination, genetic diversity

## Abstract

Rotaviruses (RVs) are important pathogens which induce gastroenteritis in different kinds of animals, including mammals and birds. Rotaviruses are divided into nine species (RVA-RVD and RVF-RVJ), and RVA-RVC and RVH can infect both humans and pigs. It is vital to understand the genetic diversity and evolution of porcine rotavirus (PoRV) for effective prevention and control of this disease. In this study, 5320 intestinal tissue samples and fecal swabs were collected from different pig farms in Guangxi Province, southern China, from 2022 to 2025. These samples were tested for PoRV species A (PoRVA), PoRVB, PoRVC, and PoRVH using the multiplex RT-qPCR. The positive samples of PoRVA were further selected to amplify and analyze the VP4, VP6, and VP7 gene sequences. The phylogenetic trees were constructed based on the PoRVA VP4, VP6, and VP7 gene sequences. Bayesian time-dynamic analysis and recombination analysis were performed for the PoRVA VP4 gene. The results indicated that the PoRVA, PoRVB, PoRVC, and PoRVH positivity rates were 16.92% (900/5320), 0.51% (27/5320), 12.71% (676/5320), and 6.22% (331/5320), respectively. Fifty-two VP4, VP6, and VP7 gene sequences were obtained from the 52 selected PoRVA-positive clinical samples. The nucleotide and amino acid identity analysis of the obtained PoRVA VP4, VP6, and VP7 genes indicated that the genetic diversity of the VP4 gene was higher than that of the VP6 and VP7 genes. The phylogenetic trees based on the VP4, VP6, and VP7 genes revealed that the predominant strains of PoRVA in Guangxi Province were the G9P[13]I5 genotype. Bayesian analysis indicated that the population size of PoRVA kept steady with no significant expansion from its discovery in the 1970s to approximately 2016, then exhibited gradual growth. Sequence analysis of the PoRVA VP4 gene revealed substitutions and recombination in the PoRVA strains, and one strain was derived from recombination of a porcine-originating strain and a human-originating strain. This study provided useful information on the molecular characteristics and genetic diversity of PoRVA and supplied important clues for in-depth research on the cross-species transmission of PoRVA.

## Simple Summary

Rotavirus species A–D and F–J can infect both humans and pigs, which characterizes gastroenteritis. In this study, porcine rotavirus (PoRV) species A (PoRVA) was tested in the intestinal tissue and fecal swab samples from Guangxi Province, southern China, from 2022 to 2025, and showed a positivity rate of 16.92% (900/5320) for PoRVA. Fifty-two positive samples were selected for amplification and sequence analysis of the VP4, VP6, and VP7 genes. The results indicated that the genetic diversity of the VP4 gene was higher than that of the VP6 and VP7 genes based on nucleotide and amino acid sequence identity analysis. The phylogenetic trees based on the VP4, VP6, and VP7 genes revealed that the predominant strains of PoRVA belonged to the G9P[13]I5 genotype. Bayesian analysis indicated that the population size of PoRVA kept steady with no significant expansion from its discovery in the 1970s to approximately 2016, then exhibited gradual growth. Sequence analysis of the VP4 gene revealed that there existed substitutions and recombination in the PoRVA strains. This study provided useful information on the molecular characteristics and genetic diversity of the circulating PoRVA strains in China.

## 1. Introduction

Rotavirus (RV), a member of the genus *Rotavirus* in the family *Sedoreoviridae*, is a non-enveloped RNA virus characterized by a double capsid structure [1]. This pathogen is spread through the fecal-oral route and causes gastroenteritis in different kinds of animals, including mammals and birds [2]. The rotavirus genome is about 18.2 kb in length and contains 11 double-stranded RNA segments encased in a three-layered concentric icosahedral symmetric capsid structure [3]. The genome encodes six structural proteins (VP1, VP2, VP3, VP4, VP6, and VP7) and five or six nonstructural proteins (NSP1, NSP2, NSP3, NSP4, and NSP5/NSP6) [3]. The core capsid consists of the VP1–VP3 proteins, the middle layer consists of the VP6 protein forming a characteristic hub-and-spoke structure, and the outermost layer consists of the antigenic determinant clusters of the viral particles, which are formed by the VP4 and VP7 proteins [3,4]. Porcine rotavirus (PoRV) infections are common in pig farms and induce gastroenteritis, which is characterized by watery diarrhea, vomiting, anorexia, and dehydration [5,6]. Recent investigations indicated that PoRV might be the causative agent of rotavirus infections in humans [7,8].

The VP4, VP6, and VP7 proteins play important roles in rotavirus infection. VP4 is a key protein in the outer capsid and consists of hemagglutination antigen and adhesion protein functional domains that determine viral pathogenicity [4,9]. The protein has hemagglutinin activity and mediates viral adsorption through C-terminal recognition of host cell receptors such as salivary acid and integrins [10]. As an important protective antigen, VP4 induces potent neutralizing antibodies and determines P genotype classification [11,12]. VP6 is the core structural protein, constituting an intermediate capsid layer that protects the viral genome and participates in the replication process [13]. As an important source of viral mRNA, VP6 maintains the structure of the transcription complex and promotes genome replication and transcription, which is essential for the maintenance of the structure and function of viral particles [14]. As a highly conserved protein, VP6 is an ideal target for diagnosis and vaccine development and has been widely used in laboratory testing, epidemiologic surveillance and classification of the rotavirus I genotype [15,16]. VP7 is essential for the stable assembly of virus particles because of its interactions with other structural proteins, such as VP4 [17]. As major neutralizing antigens, VP7-induced neutralizing antibodies are effective at blocking viral infection, and their antigenic diversity is the basis for the classification of the rotavirus G genotype [18,19]. Due to the multiple functions of the VP4, VP6, and VP7 proteins, it is vital to monitor the genetic diversity and evolutionary characteristics of rotavirus.

Rotaviruses are divided into nine species (RVA-RVD and RVF-RVJ) based on the antigenic properties and genetic diversity of the VP6 gene (https://ictv.global/report/chapter/sedoreoviridae/sedoreoviridae/rotavirus, accessed on 21 April 2025). Among them, RVA-RVC and RVH infect humans and piglets [7,8,20,21,22]. Based on the VP4 and VP7 proteins, rotaviruses can be further differentiated into subtypes, and a classification method with dual typing of P-type and G-type has been developed [23]. In 2008, the Rotavirus Classification Working Group (RCWG) extended the nucleic-acid-sequence-based nomenclature system to achieve genome-wide typing by setting a sequence similarity threshold, and the genotype nomenclature format was Gx-P[x]-Ix-Rx-Cx-Mx-Ax-Nx-Tx-Ex-Hx, which corresponds to the VP7-VP4-VP6-VP1-VP2-VP3-NSP1-NSP2-NSP3-NSP4-NSP5/6 genes [24]. To date, RVA includes at least 42 G, 58 P, 32 I, 28 R, 24 C, 24 M, 39 A, 28 N, 28 T, 32 E, and 28 H genotypes [1]; RVB includes at least 26 G, 5 P, 13 I, 5 R, 5 C, 5 M, 8 A, 10 N, 6 T, 4 E, and 7 H genotypes [25]; RVC includes at least 32 G, 28 P, 14 I, 5 R, 5 C, 5 M, 13 A, 9 N, 10 T, 8 E, and 4 H genotypes [26]; and RVH includes at least 10 G, 6 P, 6 I, 3 R, 4 C, 7 M, 6 A, 2 N, 4 T, 6 E, and 3 H genotypes [27].

Porcine RVA (PoRVA) was first reported in Australia in 1975 in diarrheic piglets [28]. PoRVA is generally considered as the most important PoRV species due to its high prevalence rate and high pathogenicity [20,29]. The diarrheic samples from the United States showed a positivity rate of 59.47% (4465/7508) for PoRVA [30,31], with the G9P[13] genotype being the most common genotype combination [32]. The 25,768 fecal samples from 230 farms in 23 provinces across China in 2022 showed an 86.52% positivity rate of PoRVA for pig farms and a 51.15% positivity rate of PoRVA for individual samples, and G9P[23]I5 was the predominant genotype combination [33]. The 1260 stool samples from the acute diarrheic piglets aged 0 to 5 weeks in Thailand during 2016–2023 showed a positivity rate of 24.0% (303/1260) for PoRVA, with G5P[23] and G4P[23] as the predominant genotypes [34]. Between 2009 and 2015, the 194 Canadian samples showed a positivity rate of 70.10% (136/194) for PoRVA [35]. Porcine RVB (PoRVB) and porcine RVC (PoRVC) were identified in diarrheic piglets in the United Kingdom and the United States in the 1980s, respectively [36,37]. PoRVB is usually coinfected with PoRVA and/or PoRVC and has a relatively low prevalence [38,39,40]. However, a Brazilian study revealed that the prevalence of PoRVB infection was as high as 71.11% (64/90), suggesting that its prevalence might be underestimated in some areas [25]. In recent years, PoRVC has spread to many countries, such as the United States, Australia, India, China, and some countries in Europe [41,42,43,44,45]. Porcine RVH (PoRVH) was first isolated from diarrheic pigs in Japan in 2011 [46] and then reported in many countries, including the United States [47], South Africa [48], Spain [49], Italy [50], China [51], Denmark [52], and so on.

The genetic diversity of rotavirus significantly increases the difficulty of vaccine development [53]. Research indicates that trivalent live attenuated vaccines utilizing combinations of dominant G/P strains conferred substantial protection against homologous strain infections, whereas bivalent vaccines (G5P[7] + G9P[7]) failed to prevent infections from heterologous strains, including G8P[1], G9P[23], and G8P[7] strains [54]. It is vital to analyze the predominant genotypes of circulating strains for accurate diagnosis and effective prevention and control of rotavirus [54,55]. In this study, the intestinal tissue samples and fecal swabs were collected from different pig farms in Guangxi Province, southern China, from 2022 to 2025, and detected PoRVA, PoRVB, PoRVC, and PoRVH using a quadruplex RT-qPCR assay [51]. Then, the PoRVA-positive samples were further selected to amplify and sequence the VP4, VP6, and VP7 genes for genetic and evolutionary analysis of PoRVA in Guangxi Province.

## 2. Materials and Methods

### 2.1. Collection of the Clinical Samples

From October 2022 to March 2025, 5320 clinical samples (453 intestinal tissue samples and 4867 fecal swab samples) were collected from 44 pig farms in 14 cities in Guangxi Province, southern China. The intestinal tissue samples came from naturally dead piglets with diarrheic symptoms, and the fecal swab samples came from piglets with diarrheic signs. They were transported to our laboratory within 6 h at ≤4 °C and stored at −80 °C until use.

### 2.2. Detection of PoRVA, PoRVB, PoRVC, and PoRVH

The intestinal tissue samples were placed in 2.0 mL EP tubes, and 1.0 mL of phosphate-buffer solution (PBS, pH 7.2, W/V = 1:5) and sterilized steel beads were added. After 3 min of oscillatory milling, 3 freeze-thaw cycles were performed, followed by centrifugation (12,000 rpm, 5 min, 4 °C), and the supernatant was collected. The fecal swabs were placed in 2.0 mL EP tubes. One mL of PBS (pH 7.2) was added, vortexed for 30 s, and freeze-thawed 3 times. Then, a centrifugation (12,000 rpm, 5 min, 4 °C) was performed to obtain the supernatant.

Two hundred µL of supernatants from tissue samples and fecal swabs were used for nucleic acid extraction using the MiniBEST Viral DNA/RNA Nucleic Acid Extraction Kit Ver.5.0 (TaKaRa, Dalian, China), and tested for PoRVA, PoRVB, PoRVC, and PoRVH using a quadruplex RT-qPCR assay previously developed in our laboratory [51]. Briefly, the specific primers and probes as follows were used: forward primer (RVA-VP6-F) AATATGACACCAGCAGTTGCAAA, reverse primer (RVA-VP6-R) ACAGATTCACAAACTGCAGATTCAA and probe (RVA-VP6-P) CY5-CAAGCACCGCCATTTATATTTCATGCTACA-BHQ3 for PoRVA; forward primer (RVB-VP6-F) GTGTCYGCRTWTGCTGC, reverse primer (RVB-VP6-R) CCTYTCGAAGCACTYCC and probe (RVB-VP6-P) VIC-GGRAGCTGACGCCGGATCAGA-BHQ1 for PoRVB; forward primer (RVC-VP6-F) GTGAAGAGAATGGTGATGTAG, reverse primer (RVC-VP6-R) GTTCACATTTCATCCTCCTG and probe (RVC-VP6-P) FAM-TAGCATGATTCACGAATGGGTTTAG-BHQ1 for PoRVC; and forward primer (RVH-VP6-F) GGAAGAGCTACTGGAAAGATGG, reverse primer (RVH-VP6-R) GACTCCTGAGCATGGTACTTTC and probe (RVH-VP6-P) Texas Red-CAGTTCAAGGCAGACCAGGAGGAA-BHQ2 for PoRVH. The reaction system in a total volume of 20 µL included 2× One Step RT-PCR Buffer III (TaKaRa, Dalian, China) 10 μL, Ex Taq HS (TaKaRa, Dalian, China) (5 U/µL) 0.4 μL, PrimeScript RT Enzyme Mix II (TaKaRa, Dalian, China) 0.4 μL, RVA-VP6-F/RVA-VP6-F/RVA-VP6-P (20 μM) 0.1 μL each, RVB-VP6-F/RVB-VP6-R (20 μM) 0.3 μL each, RVB-VP6-P (20 μM) 0.1 μL, RVC-VP6-F/RVC-VP6-R (20 μM) 0.3 μL each, RVC-VP6-P (20 μM) 0.1 μL, RVH-VP6-F/RVH-VP6-F/RVH-VP6-P (20 μM) 0.1 μL each, total nucleic acid 2.0 μL, and distilled water up to 20.0 μL. The one-step amplification parameters were as follows: 42 °C 5 min, 95 °C 10 s, and then 40 cycles of 95 °C 5 s, 58 °C 34 s. The samples with a Ct value ≤ 36 were considered as positive samples.

### 2.3. Amplification and Sequencing of the PoRVA VP4, VP6, and VP7 Genes

After detection, 52 PoRVA-positive samples were selected for sequence analysis. The criteria for selecting the positive samples were that they came from different cities on different dates with Ct values ≤ 25 cycles. Total RNA was extracted, reverse-transcribed into cDNA with the PrimeScript™ II 1st Strand cDNA Synthesis Kit (TaKaRa, Dalian, China), and used for amplification and sequencing of the PoRVA VP4, VP6, and VP7 genes.

The specific primers were designed to amplify the PoRVA VP4, VP6, and VP7 genes, referencing the PoRVA genome sequences obtained from the National Center for Biotechnology Information (NCBI) (https://www.ncbi.nlm.nih.gov/nucleotide/, accessed on 10 September 2022) (Table 1). The PCR amplification system included 25 μL of Premix Taq Mix (Ex Taq Version 2.0 with dye) (TaKaRa, Dalian, China), 1 μL of forward and reverse primer each (20 μM), 5 μL of cDNA, and distilled water to a final volume of 50 μL. The amplification protocol was as follows: for the VP4A gene fragment, 95 °C 4 min, 36 cycles of 95 °C 42 s, 55 °C 30 s, and 72 °C 75 s, followed by 72 °C 12 min; for the VP4B, VP4C, VP6 and VP7 gene fragments, 95 °C 4 min, 36 cycles of 95 °C 42 s, 52 °C 30 s, and 72 °C 70 s, followed by 72 °C 12 min.

The PCR products were purified using the MiniBEST DNA Fragment Purification Kit (TaKaRa, Dalian, China), ligated into the pMD18-T vector (TaKaRa, Dalian, China), and subsequently transformed into *E. coli* DH5α-competent cells (TaKaRa, Dalian, China). The positive clones were inoculated in LB medium supplemented with ampicillin, cultured at 37 °C for 20–24 h, and sequenced by IGE Biotechnology Ltd. (Guangzhou, China). The obtained gene fragment sequences were spliced using the EditSeq function in Lasergene DNAstar 7.0 software (https://www.dnastar.com/software/, accessed on 12 April 2025) to obtain the complete VP4, VP6, and VP7 gene sequences, which were further confirmed using the BLAST program at NCBI (https://blast.ncbi.nlm.nih.gov/Blast.cgi, accessed on 12 April 2025).

### 2.4. Homology and Phylogenetic Analysis of the PoRVA VP4, VP6, and VP7 Genes

The reference sequences, including 58 VP4, 50 VP6, and 62 VP7 gene sequences of PoRVA (Supplementary Table S1), were downloaded from NCBI (https://www.ncbi.nlm.nih.gov/nucleotide/; accessed on 21 April 2025). The reference strains originated from different countries, such as China, the USA, Uganda, Belgium, Japan, Russia, Italy, Germany, Thailand, Vietnam, Spain, and Korea. The VP4, VP6, and VP7 gene sequences obtained in this study (Supplementary Table S2) were analyzed together with the reference gene sequences using the Clustal W method within DNAstar 7.0 software (https://www.dnastar.com/software/; accessed on 21 April 2025). The phylogenetic trees were constructed using the MEGA 12.0.11 software (https://megasoftware.net/; accessed on 21 April 2025), and optimized using the Interactive Tree of Life (iTOL) (https://itol.embl.de/; accessed on 21 April 2025).

### 2.5. Bayesian Temporal Dynamics Analysis of the PoRVA VP4 Gene

The 52 obtained PoRVA VP4 sequences (Supplementary Table S2) and the 36 reference sequences downloaded from NCBI (Supplementary Table S3) were aligned using the MEGA 12.0.11 software (https://megasoftware.net/, accessed on 21 April 2025). The suitable substitution models were calculated using the ModelFinder tool of PhyloSuite v1.2.2 software (http://phylosuite.jushengwu.com, accessed on 21 April 2025). The relaxed molecular clock, the GTR+F+G4 substitution model and the Bayesian SkyGrid model were finally selected, followed by 200 million steps of Markov chain Monte Carlo (MCMC) in parallel on 3 chains with a burn-in of 10%. The data after the run were visualized using the Tracer v1.6 software (https://beast.community/tracer, accessed on 21 April 2025) to acquire the gene evolutionary rate, and the convergence of all the parameters was visually confirmed, with ESS > 200 considered valid for the parameters. The data were computed using the Tree Annotator v1.10.4 (https://beast.community/treeannotator, accessed on 21 April 2025) to acquire the maximum clade confidence (MCC) tree, which was annotated after 10% aging and visualized using FigTree version 1.4.5 (https://beast.community/figtree, accessed on 21 April 2025).

### 2.6. Recombination Analysis of the PoRVA VP4 Gene

The VP4 gene sequences of the 52 obtained PoRVA strains (Supplementary Table S2) were compared with those of the 58 reference strains (Supplementary Table S1) using the BioEdit v7.2.5 software (https://www.bioedit.com/, accessed on 21 April 2025). All sequences were analyzed for recombination using the Recombination Detection Program (RDP4) software (https://health.uct.ac.za/computational-biology, accessed on 21 April 2025). Seven methods (RDP, Chimaera, SiScan, BootScan, GENECONV, MaxChi, and 3Seq) were used to detect recombination events and breakpoints per the recommendations of the RDP manual. The sequences supported by at least six algorithms were considered potential recombination sequences. Then, the putative recombination events were further verified using the SimPlot v3.5.1 software (https://github.com/Stephane-S/Simplot_PlusPlus, accessed on 21 April 2025).

### 2.7. Amino Acid Sequence Analysis of the PoRVA VP4 Gene

To analyze the genetic identity between different genotypes, the amino acid sequences of the PoRVA VP4 gene were analyzed using the BioEdit v.7.2.5 software (https://bioedit.com/, accessed on 21 April 2025).

## 3. Results

### 3.1. Detection Results of PoRV in the Clinical Samples

**Figure 1:**
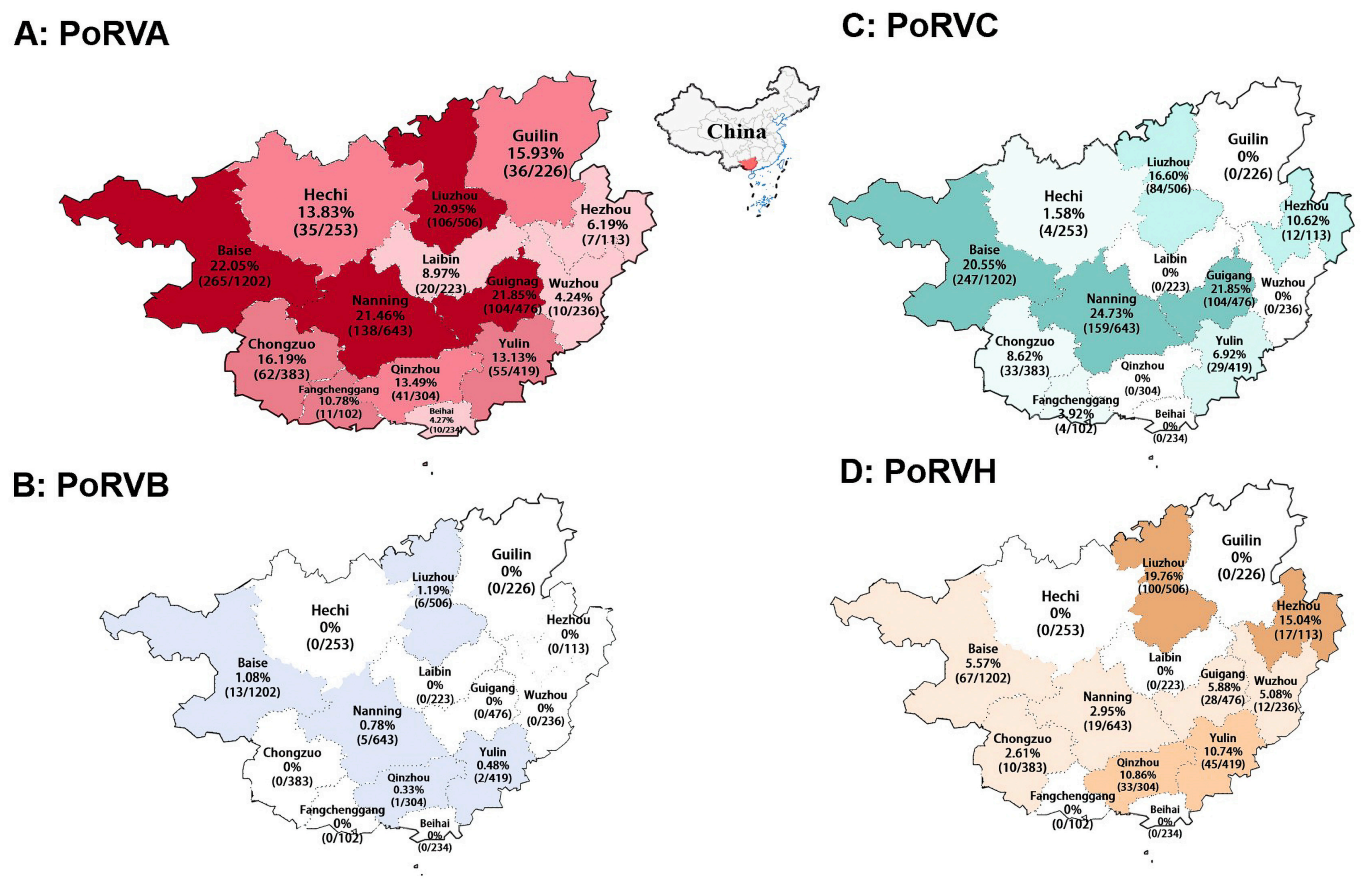
The distribution of PoRVA (**A**), PoRVB (**B**), PoRVC (**C**), and PoRVH (**D**) positive samples in Guangxi Province during 2022–2025.

The 5320 clinical samples from Guangxi Province, southern China, were tested for PoRV using a previously developed quadruplex RT-qPCR assay [51]. The positivity rates of PoRVA, PoRVB, PoRVC, and PoRVH were 16.92% (900/5320), 0.51% (27/5320), 12.71% (676/5320), and 6.22% (331/5320), respectively, showing significant difference among different species of PoRVA (chi-square analysis, *p* ≤ 0.05) (Supplementary Table S4). The positivity rates in small intestine and feces were 14.32% and 17.31% for PoRVA (*p* ≤ 0.05), 0.49% and 0.51% for PoRVB (*p* > 0.05), 9.91% and 13.15% for PoRVC (*p* ≤ 0.05), and 5.75% and 6.28% for PoRVH (*p* > 0.05), respectively, showing the positivity rates of PoRVA and PoRVC were significantly higher in fecal swab samples than in tissue samples (Supplementary Table S5). In brief, PoRVA-positive samples were found in all 14 cities in Guangxi Province, with the highest positivity rate of 22.05% in Baise city, and positivity rates higher than 20% were found in Nanning, Liuzhou, and Guigang. PoRVB was found only in Louzhou, Baise, Nanning, Yulin, and Qinzhou cities, with relatively low positivity rates of less than 2%. PoRVC had the highest positivity rate of 24.73% in Nanning city, and no positive sample was detected in Guilin, Beihai, Laibin, Qinzhou, or Wuzhou. PoRVH was found in Nanning, Liuzhou, Chongzuo, Hezhou, Guigang, Baise, Yulin, Qinzhou and Wuzhou, with the highest positivity rate of 19.76% in Liuzhou city. The distributions of the PoRVA, PoRVB, PoRVC, and PoRVH-positive samples from Guangxi Province are shown in Figure 1. In addition, co-infections of PoRVA, PoRVB, PoRVC, and PoRVH were also detected in the clinical samples, as shown in Table 2.

### 3.2. Acquirement of the PoRVA VP4, VP6, and VP7 Gene Sequences

Based on different sampling times and locations, and detection Ct values ≤ 25 of the samples, 52 PoRVA-positive samples were selected for VP4, VP6, and VP7 gene amplification and sequencing. Finally, 52 VP4, 52 VP6, and 52 VP7 gene sequences were acquired and uploaded to the NCBI GenBank database (https://www.ncbi.nlm.nih.gov/nucleotide/, accessed on 21 April 2025) under the following accession numbers (Supplementary Table S2): PV655424-PV655475 for the 52 VP4 gene sequences, PV655191-PV655242 for the 52 VP6 gene sequences, and PV655243-PV655294 for the 52 VP7 gene sequences.

### 3.3. Homology of the PoRVA VP4, VP6, and VP7 Genes

The obtained PoRVA VP4, VP6, and VP7 gene nucleotide and deduced amino acid sequences (Supplementary Table S2), together with those of the reference strains (Supplementary Table S1), were analyzed using the Clustal W method in BioEdit v7.2.5 software (https://www.bioedit.com/, accessed on 21 April 2025). The results are shown in Table 3. The obtained 52 PoRVA strains showed 67.9–100.0% nucleotide identity and 72.1–100.0% amino acid identity within the obtained strains and showed 60.8–98.7% nucleotide identity and 63.0–99.6% amino acid identity with the reference strains. The VP4 gene showed the highest genetic diversity.

### 3.4. Phylogenetic Analysis Based on the PoRVA VP4, VP6, and VP7 Gene Sequences

**Figure 2:**
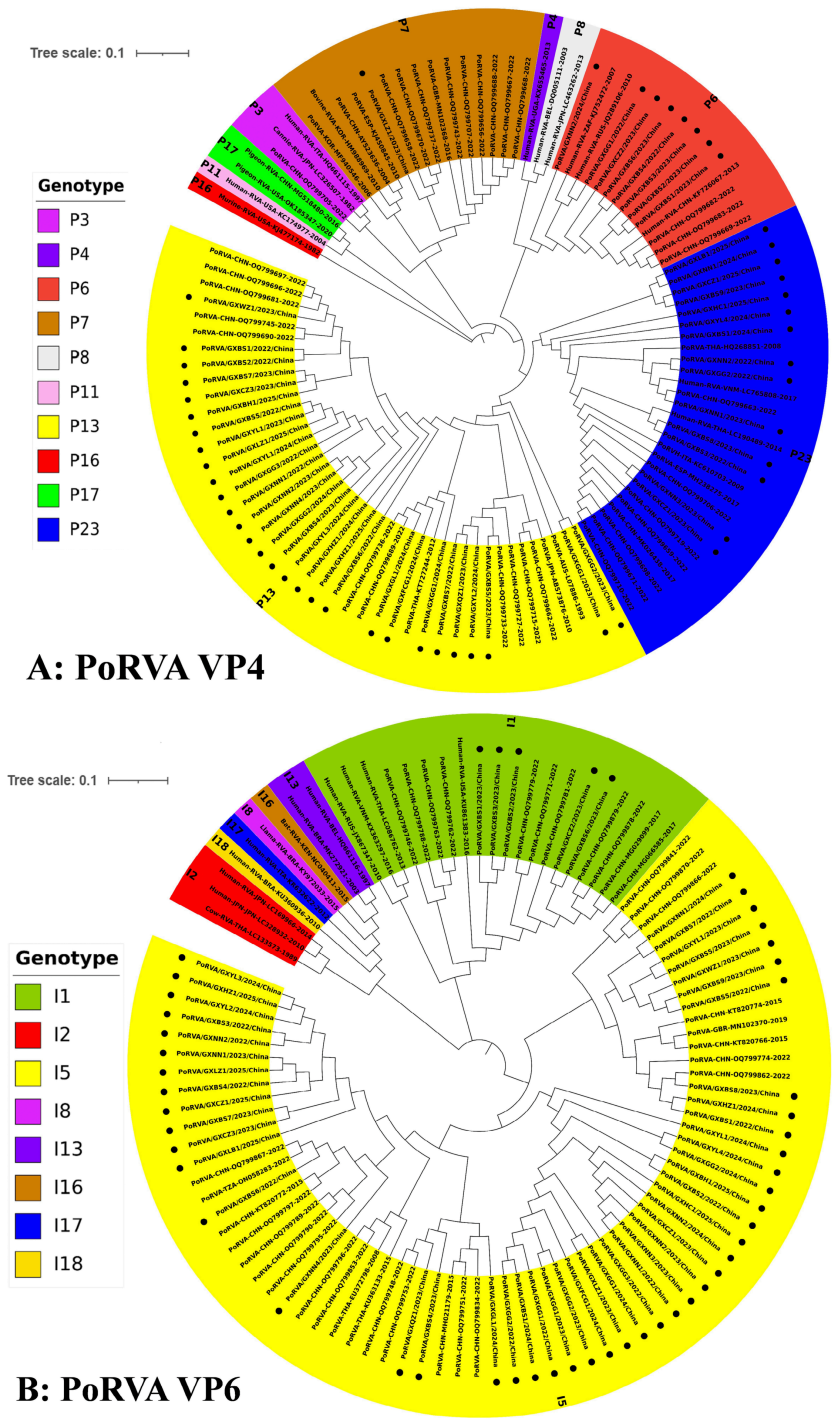

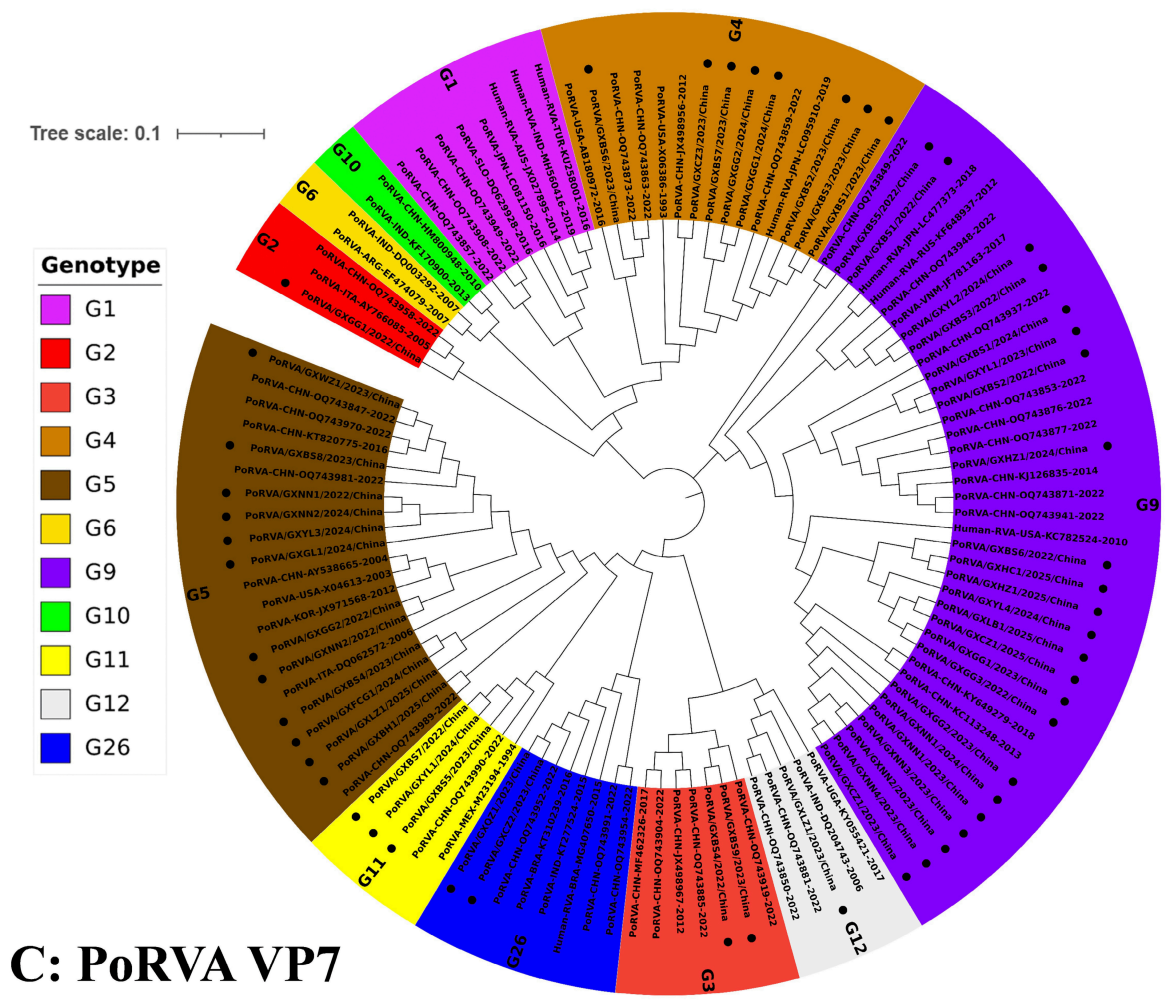
Phylogenetic trees based on the PoRVA VP4 (**A**), VP6 (**B**), and VP7 (**C**) gene nucleotide sequences. The black spots represent the gene sequences obtained in this study.

To analyze the genetic characteristics of PoRVA, the 52 obtained VP4 gene sequences and the 58 reference gene sequences were used to construct a phylogenetic tree (Figure 2A). The results revealed that the 52 obtained PoRVA strains were clustered into four different genotype branches. Among them, 30 strains (30/52, 57.69%) originating from 10 cities in Guangxi Province were clustered into the P[13] branch, which also contained reference strains from China, Japan and Australia. In addition, of the other 22 strains, 14 strains belonged to the P[23] genotype, 7 strains belonged to the P[6] genotype, and one strain belonged to the P[7] genotype.

The 52 obtained VP6 gene sequences and the 58 reference gene sequences were utilized to construct a phylogenetic tree (Figure 2B). The results revealed that the 52 obtained strains were clustered into two genotypes: 47 strains (47/52, 90.38%) of genotype I5, which also included reference strains from China, the United Kingdom and Tanzania, and five strains of genotype I1.

The 52 obtained VP7 gene sequences and the 62 reference gene sequences were utilized to construct a phylogenetic tree (Figure 2C). The results revealed that the 52 obtained strains were clustered into eight genotypes: one G2 strain, two G3 strains, eight G4 strains, twelve G5 strains, 23 G9 strains, three G11 strains, one G12 strain, and two G26 strains. Of the obtained strains, the G9 genotype (23/52, 44.23%) was the predominant strain, and the G26 genotype branch included both human-derived and porcine-derived rotavirus strains.

### 3.5. Genetic Evolutionary Rates of the PoRVA VP4, VP6, and VP7 Genes

The evolutionary rates of the VP4, VP6, and VP7 genes of the PoRVA strains from Guangxi Province and the reference strains were analyzed using the BEAST v1.10.4 software (https://beast.community, accessed on 21 April 2025). The results demonstrated that the PoRVA VP4, VP6, and VP7 genes had the evolutionary rates of 4.09 × 10^−3^ (95% HPD: 2.55 × 10^−3^–5.60 × 10^−3^) substitution/site/year (s/s/y), 1.49 × 10^−3^ (95% HPD: 9.14 × 10^−4^–2.04 × 10^−3^) s/s/y, and 1.39 × 10^−3^ (95% HPD: 8.69 × 10^−4^–1.95 × 10^−3^) s/s/y, respectively, showing that the VP4 gene presented the highest evolutionary rate.

### 3.6. Bayesian Time Dynamic Analysis of the PoRVA VP4 Gene

**Figure 3:**
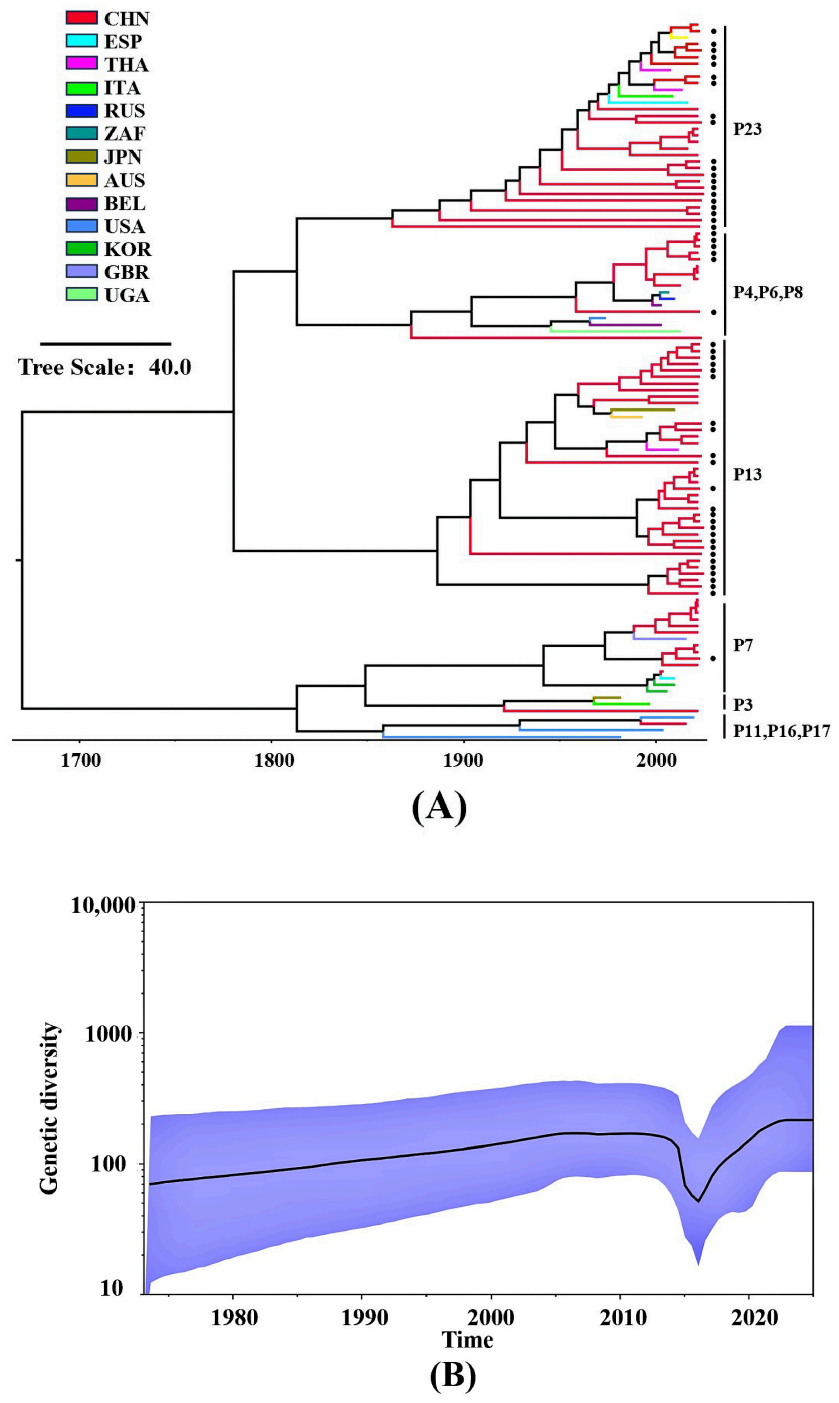
(**A**) MCC tree constructed using the PoRVA VP4 gene nucleotide sequences. The obtained sequences in this study are denoted by black dots. (**B**) Bayesian skyline of the PoRVA VP4 gene. The dark purple line represents the mean genetic diversity, whereas the light purple shading denotes a 95% CI. CHN: China; ESP: Kingdom of Spain; THA: Thailand; ITA: Italy; RUS: Russia; ZAF: Republic of South Africa; JPN: Japan; AUS: Australia; BEL: Belgium; USA: the United States of America; KOR: Korea; GBR: Great Britain; UGA: Uganda.

The time-scale analysis of the MCC tree of the PoRVA VP4 gene was constructed using the BEAST v1.10.4 software (https://beast.community, accessed on 21 April 2025) and FigTree version 1.4.5 (https://beast.community/figtree, accessed on 21 April 2025). The results indicated that the obtained PoRVA strains (Supplementary Table S2) and the reference strains (Supplementary Table S3) were clustered into 10 evolutionary branches, and the strains from Guangxi Province were divided into four branches. The branch distribution pattern was highly consistent with the phylogenetic tree of the PoRVA VP4 gene constructed in this study (Figure 3A).

The effective population dynamics of the PoRVA VP4 gene based on Bayesian skyline analysis is shown in Figure 3B. The dynamics of the effective population size showed obvious stage-specific characteristics. Between 1975 and 2014, the population size remained relatively stable; a significant decline in population size was observed between 2014 and 2016. Since 2016, the population size has begun to expand and has exceeded the pre-2014 level post-2020. Overall, the PoRVA in Guangxi Province showed a significant trend toward population expansion.

### 3.7. Recombination of the PoRVA VP4 Gene Sequence

**Figure 4:**
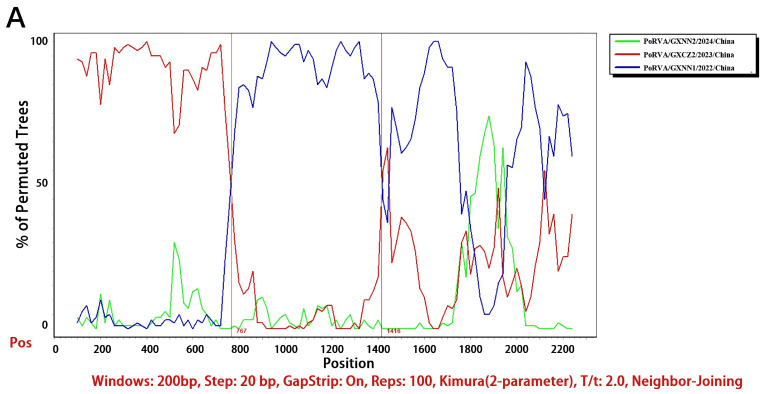

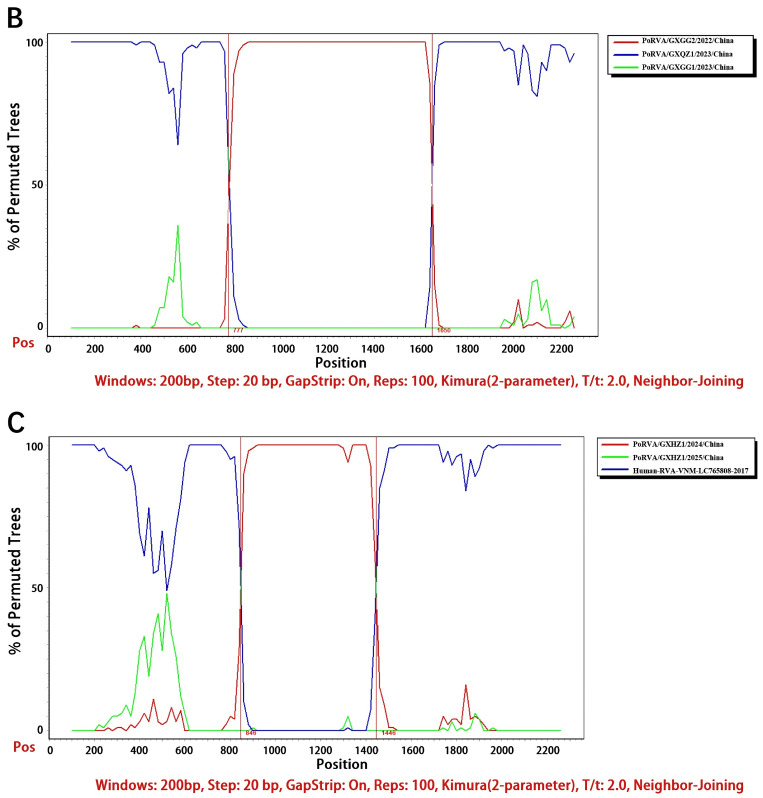
Recombination analysis of the PoRVA/GXNN2/2024/China strain (**A**), PoRVA/GXGG1/2023/China strain (**B**), and PoRVA/GXHZ1/2025/China strain (**C**).

Recombination analysis of the PoRVA VP4 gene of the 52 obtained strains and the 58 reference strains was performed using the Recombination Detection Program (RDP4) software (https://health.uct.ac.za/computational-biology, accessed on 21 April 2025). Seven detection algorithms of RDP, GENECONV, BootScan, MaxChi, Chimaera, 3Seq, and SiScan were applied, and all the results were validated using the Simplot v3.5.1 software (https://github.com/Stephane-S/Simplot_PlusPlus, accessed on 21 April 2025). The results indicated that three potential recombination events were identified: (1) The PoRVA/GXNN2/2024/China strain originated from the recombination between the PoRVA/GXCZ2/2023/China strain (primary parent) and the PoRVA/GXNN1/2022/China strain (secondary parent), with a recombination region of 767 nt–1416 nt, showing 99.6% similarity with the primary parent and 99.9% similarity with the secondary parent. (2) The PoRVA/GXGG1/2023/China strain was derived from recombination between the PoRVA/GXQZ1/2023/China strain (primary parent) and the PoRVA/GXGG2/2022/China strain (secondary parent), with a recombination region of 777 nt–1650 nt, showing 98.5% similarity with the primary parent and 94.7% similarity with the secondary parent. (3) The PoRVA/GXHZ1/2025/China strain was derived from recombination between the PoRVA/GXHZ1/2024/China strain (primary parent) and the Human-RVA-VNM-LC765808-2017 strain (secondary parent), with a recombination region of 846–1446 nt, showing 98.9% similarity with the primary parent and 95.3% similarity with the secondary parent (Figure 4).

### 3.8. Analysis of the Amino Acid Sequence of the PoRVA VP4 Gene

**Figure 5:**
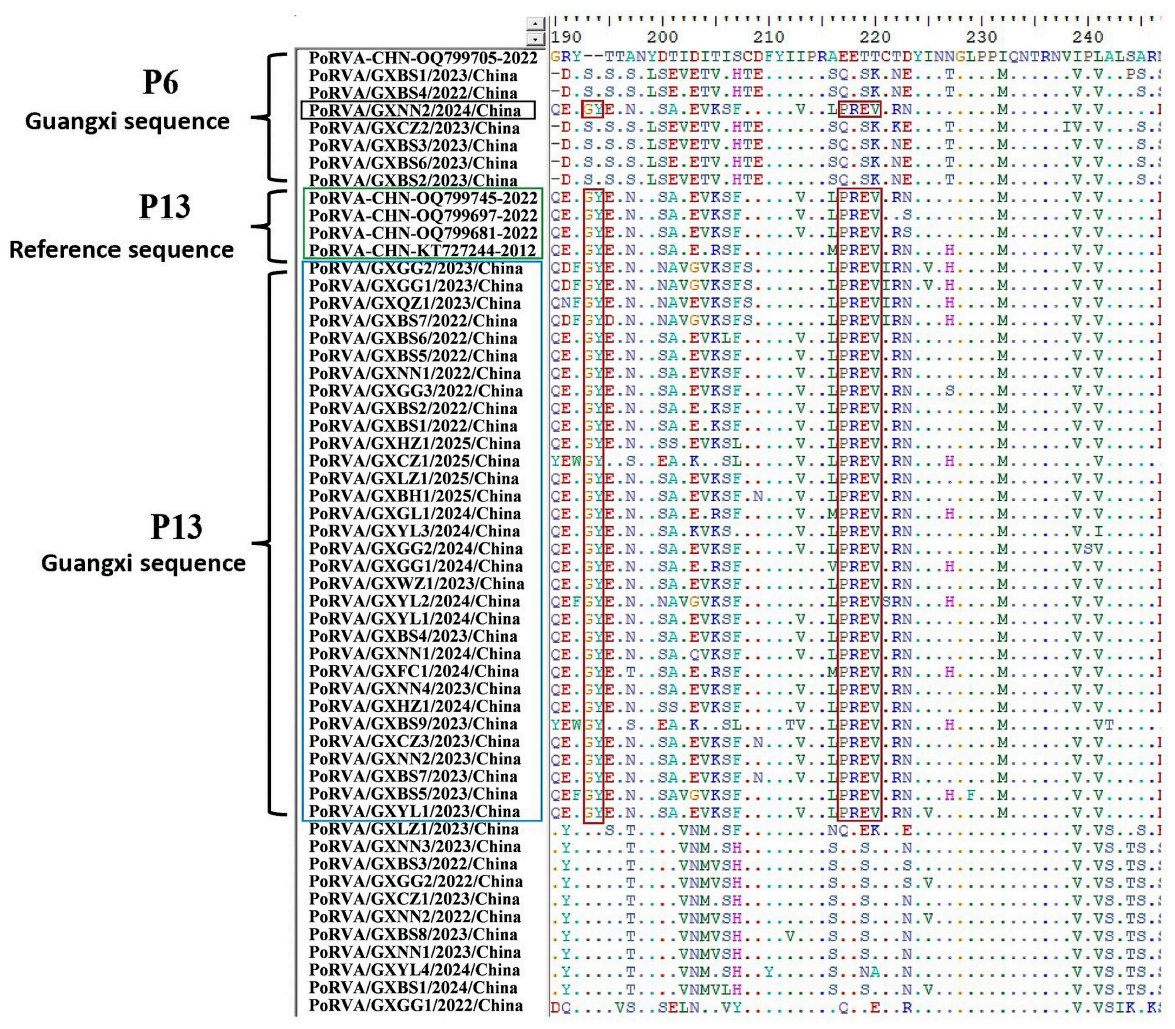
The amino acid sequence analysis of the PoRVA VP4 gene. Partial amino acid substitution of the PoRVA VP4 gene is shown.

The amino acid sequences of the 52 obtained PoRVA VP4 genes were systematically compared with those of other genotypes using the BioEdit v7.2.5 software (https://bioedit.com/, accessed on 21 April 2025). The results revealed that the P[13] strains presented unique molecular features at several sites: there were two specific amino acid insertion at sites 193–194 (193G,194Y); there were multiple amino acid substitutions at sites 216–220; and a P[6] strain (PoRVA/GXNN2/2024/China), which originated from recombination of the P[13] strain with the P[6] strain, presented all the abovementioned molecular features (Figure 5).

## 4. Discussion

Rotavirus infection can induce severe diarrhea syndrome in young animals, especially piglets. It not only leads to intestinal epithelial cell damage but also destroys the intestinal mucosal immune barrier, which significantly increases the risk of secondary infection [56]. PoRVA, PoRVB, PoRVC, and PoRVH have been reported worldwide and have induced significant economic losses to the livestock industry [29,30,31,32,33,34,35,36,37,38,39,40,41,42,43,44,45,46,47,48,49,50,51,52]. Of these four pathogens, PoRVA is widely circulating in pig farms worldwide and restricts the healthy development of the pig industry; therefore, it has become the most concerning pathogen among these four pathogens [31,32,33,34,35,36,57,58]. Especially, mixed infections exacerbate the clinical signs and pathological damage induced by these pathogens [51,52,59,60]. Rotaviruses were detected in 81.11% of the Danish pigs, with different positivity rates of PoRVA (37.78%), PoRVB (38.89%), PoRVC (28.89%) and PoRVH (1.11%) [52]. The clinical samples from Guangxi Province during 2021–2022 showed high positivity rates of PoRVA (42.71%, 618/1447), PoRVB (26.95%, 390/1447), PoRVC (42.92%, 621/1447), and PoRVH (13.68%, 198/1447), and I5 was the predominant genotype [51]. The fecal samples from suckling diarrheic piglets from 112 pig farms in Brazil during 2015–2021 showed 9.20% (47/511), 19.96% (102/511), 12.72% (65/511), and 2.15% (11/511) for PoRVA, PoRVB, PoRVC, and PoRVH, respectively [61]. These indicated that porcine rotaviruses are widespread and complex, and it is necessary to investigate the epidemic situation, predominant genotype, and genetic characteristics of them in China.

In this study, the clinical samples from Guangxi Province showed positivity rates of 16.92%, 0.51%, 12.71%, and 6.22% for PoRVA, PoRVB, PoRVC, and PoRVH, respectively. Compared with those of PoRVA, the positivity rates of PoRVB, PoRVC and PoRVH were relatively low, but the threat they posed to the pig industry still cannot be ignored. The different positivity rates of PoRV were shown in different cities (Supplementary Table S4), which might be attributed to the different breeding habits, pig trade flows, and vaccination programs adopted across Guangxi Province. Overall, different species of PoRV have also been reported in different provinces in China. PoRVA showed positivity rates of 28.76% in Shandong province [56], 4.29% in Heilongjiang province [62], 17.59% in nine different provinces across China [63], and 51.15% in 23 provinces across China [33]. PoRVB showed a positivity rate of 26.95% in Guangxi province [51] and an 83.33–100% positivity rate of PoRVB was detected in the diarrheic piglets from three affected farms in the Anhui, Liaoning, and Jilin provinces of China [64]. PoRVC showed positivity rates of 4.35% in Zhejiang province [65]; 18.68% in Zhejiang, Shandong, Jiangsu, and Shanxi provinces [66]; and 11.36% in Jiangsu province [67]. PoRVH showed a positivity rate of 13.68% in Guangxi province [51]. These findings confirmed that porcine rotaviruses are widespread across China. More attention should be paid to their epidemic situations, and more effective measures should be proposed.

Of the 52 obtained PoRVA strains in this study, the nucleotide and amino acid sequence identities were 67.91–99.96% and 72.12–100% for the VP4 gene, 81.24–99.92% and 83.25–100% for the VP6 gene, and 73.16–100% and 76.54–100% for the VP7 gene. This indicated that the VP4 gene showed the highest genetic variability. In addition, the evolutionary rate of the VP4 gene (4.09 × 10^−3^ s/s/y) was higher than that of the VP6 gene (1.49 × 10^−3^ s/s/y) and the VP7 gene (1.39 × 10^−3^ s/s/y), suggesting that the VP4 gene showed a higher evolutionary rate and higher genetic variety. Other papers have also reported the evolutionary rate of rotaviruses. RVA G6 strains in calves showed an evolutionary rate of 1.24 × 10^−3^ s/s/y for the VP7 gene sequences [68]. The VP4 gene sequences of RVA P[6] genotype from 13 countries in Africa had 1.05 × 10^−3^ s/s/y [69]. The VP7 gene sequences showed an evolutionary rate of 7.279 × 10^−4^ s/s/y for RVA G3 genotype strains [70], 8.869 × 10^−4^ s/s/y for RVA G1 genotype strains [71], 1.63 × 10^−3^ s/s/y for RVA G9 genotype strains [72], and 1.678 × 10^−3^ s/s/y for G12 genotype strains [73]. The NSP4 gene of G2 genotype strains showed an evolutionary rate of 1.2 × 10^−3^ s/s/y [74]. These indicated that porcine rotaviruses circulating in pig herds exhibited genetic diversity with different evolutionary rates in different genes.

Bayesian skyline model plots indicated that the effective population size of PoRVA remained relatively stable between 1975 and 2014 but showed a significant decrease from 2014 to 2016, which might be related to the widespread use of rotavirus vaccines during this period [54,61]. In this study, sustained expansion in population size has been observed since 2016, which is greater than the pre-2014 population size, suggesting that vaccination selection pressures may have driven the adaptive evolution of PoRVA [6,75].

PoRVA primarily comprises 12 G and 16 P genotypes. The G3, G4, G5, G9, and G11 were recognized as the predominant G genotypes in swine, which are typically correlated with P[5], P[6], P[7], P[13], and P[28] [6,24]. The genotyping results of the PoRVA VP7, VP4, and VP6 genes in Guangxi Province revealed that G9, P[13] and I5 were the predominant genotypes in pig herds, respectively; the P[13] and I5 genotypes were found in 12 prefecture-level cities. In terms of genotype combinations, G9P[13]I5 was the most predominant strain, with a 32.69% (17/52) detection rate. The less prevalent strains included G5P[13]I5 (15.38%, 8/52) and G9P[23]I5 (11.54%, 6/52) genotypes. These three genotype combinations accounted for 59.62% of all the tested samples, which was similar to the results of a previous report [33]. These results provide useful information on the circulating PoRV in Guangxi Province, southern China. The reasons that genotype G9P[13]I5 but not genotype G9P[13]I5 (the predominant genotype in North China) is the predominant genotype in Guangxi Province might be attributed to the high humidity and heat climate conditions in subtropical regions, unique breeding and management practices, and its relatively independent closed-loop breeding system. In addition, the circulating porcine rotaviruses might include different genotypes. The PoRVA strains from 230 farms in 23 provinces of China indicated the predominant genotype combination of G9P[23]I5. The VP7 G9 genotype constituted 56.55% of the obtained strains; the VP4 genotypes were determined as P[13] (42.22%), P[23] (25.56%) and P[7] (22.22%); and the VP6 gene exhibited I5 (88.81%) and I1 (11.19%) genotypes [33]. The clinical samples from 29 provinces of China in 2022 showed a 44.00% (3957/8588) positivity rate of PoRVA, and the G9, G5, and G4 genotypes were the predominant genotypes [76]. The 1791 diarrhea samples from five provinces in southern China during 2021–2023 showed the prevalence rates of PoRV ranging from 25.81% to 50.81%, and G9 was the dominant genotype, followed by the G5, G3 and G4 genotypes [77]. The clinical samples from East China had a 16.83% positivity rate for PoRVA, and the nine obtained PoRVA strains belonged to G9P[7] (5/9), G5P[13] (2/9), G9P[13] (1/9), and G5P[7] (1/9), respectively [78]. Since the potential cross-protection between rotavirus strains with different genotypes is limited [6,53,54,79], the application of vaccines based on prevalent genotypes has become critical for effective vaccination. At present, the genotypes of the officially approved commercial vaccine strains include PoRVA NX strain (Genotype G5P[7]), JS01-R strain (Genotype G9P[23]), NJ2012 strain (Genotype G9P[7]), AHFY2022 strain (Genotype G9P[23]), GD2024 strain (Genotype G5P[13], and JS2024 strain (Genotype G9P[23]). In this study, the distribution characteristics of the major prevalent genotypes of PoRVA in China was systematically analyzed and confirmed that genotypes G9P[13]I5 (32.69%), G5P[13]I5 (15.38%) and G9P[23]I5 (11.54%) are the main genotypes circulating in pig herds, which is similar to the situation in Guangdong Province, another province in southern China [55]. This information provided a useful basis for the application of multivalent vaccines against the dominant strains. That is to say, the bivalent inactivated vaccine GD2024+JS2024 strains (G5P[13]+G9P[23] genotypes), specifically designed for the circulating strains in South China and currently available on the Chinese market, is the preferred vaccine in southern China.

RVA strains show genetic diversity and have zoonotic potential. To date, at least 10 G genotypes (G1–G5, G9–G12, and G26) and seven P genotypes (P[4], P[6], P[8], P[13], P[14], P[19], and P[25]) of porcine origin have been identified in humans [6]. It was reported that some PoRVA strains shared high genomic identity with human-derived RVA strains, and interspecies transmission and recombination might have occurred [7,8,21,22]. In this study, the phylogenetic analysis based on the VP4 gene revealed that some PoRVA strains showed relatively high homology with human-origin RVA strains; both the P[23] and P[6] branches contained porcine and human rotavirus strains. The phylogenetic analysis based on the VP7 gene revealed that the G26 genotype branch included both human-originated and swine-originated rotavirus strains. These results indicate that some genotypes of PoRVA strains had high genomic homology with human strains. Recombination analysis in this study found that the PoRVA strain PoRVA/GXHZ1/2025/China was generated by recombination between the porcine-derived strain (PoRVA/GXHZ1/2024/China) and the human-derived strain (Human-RVA-VNM-LC765808-2017), highlighting the dynamic interaction between human and porcine rotaviruses. In addition, recombination events have also been reported in other genes. A natural recombinant event was found in the VP7 and VP3 genes in the PoRVA strain [80]. Two PoRVA strains, RVA/pig-wt/ZMB/LSK0137 (G4P[6]I5 genotype) and RVA/pig-wt/ZMB/LSK0147 (G9P[13]I5 genotype) from Zambia, exhibited possible reassortment and interspecies transmission of RVA involving pigs and humans [81]. The reassortment and potential risk of interspecies transmission of PoRV remind us to pay more attention to their prevention and control in order to reduce their harm to animals and humans.

Some limitations of this study should be considered. Firstly, the 52 positive samples were selected for sequence analysis based on Ct value (≤25), sampling time, and geographic location. The samples with low Ct values (≤25) might contain high viral loads, which ensures obtaining enough PCR products of the target fragments for gene sequencing. The samples from different sampling times and geographic locations were selected to ensure the representativeness of the samples and further ensure that the obtained sequences reflect the true situation as much as possible. However, this strategy might introduce sampling bias and overlook genetically important strains with lower viral loads. Secondly, the genetic diversity and evolution of PoRVA were analyzed based on the VP4, VP6, and VP7 genes. If the whole-genome sequences of PoRVA were obtained and used for sequence analysis, it would provide a more comprehensive understanding of viral evolution and reassortment of the circulating strains in China. Since rotavirus evolution is largely driven by reassortment among all 11 genome segments, limiting the analysis to three genes might restrict the conclusions regarding viral evolution. Therefore, the whole-genome sequences should be considered in further studies. Thirdly, this study focused on the genetic diversity and evolution of PoRVA in Guangxi Province due to the high positivity rate of PoRVA in the clinical samples. Due to the widespread circulation and significant importance of PoRVB, PoRVC, and PoRVH to the pig industry, further study on the genetic characteristics and diversity of these rotavirus species is necessary in order to perform effective prevention and control measures.

## 5. Conclusions

In this study, the 5320 clinical samples from Guangxi Province, southern China, during 2022–2025 showed positivity rates of 16.92%, 0.51%, 12.71%, and 6.22% for PoRVA, PoRVB, PoRVC, and PoRVH, respectively, indicating the widespread prevalence of PoRV in different pig farms. The predominant strains of PoRVA in Guangxi Province had the G9P[13]I5 genotype. The PoRVA VP4 gene showed a higher evolutionary rate and genetic diversity than that of the VP6 and VP7 genes. The population size of PoRVA was relatively stable from its discovery in 1975 until 2016 and then continued to exhibit gradual growth. The PoRVA VP4 gene showed substitution and recombination in the PoRVA strains, and one strain might have originated from the potential recombination between porcine-derived and human-derived strains. This study provides important information on the molecular characteristics and genetic diversity of PoRVA in southern China.

## Figures and Tables

**Table 1:** Primers used to amplify the PoRVA VP4, VP6, and VP7 genes.

Gene	Primer	Sequence (5′→3′)	Product/bp
PoRVA-VP4	PoRVA-VP4A-F	GGCTATAAAATGGCTTCACTC	1103
	PoRVA-VP4A-R	CCATATTTCTGAATGCTTGAGAATC	
	PoRVA-VP4B-F	TGGAAAGAGATGCAATATAACAGAG	809
	PoRVA-VP4B-R	AACATWGAAAACATATCTAATGG	
	PoRVA-VP4C-F	TATCARACACCAATTATGAATTC	916
	PoRVA-VP4C-R	GGTCACAACCTCTAGACACTACTTACA	
PoRVA-VP6	PoRVA-VP6-F	GGCTTTTAAACGAAGTCT	1315
	PoRVA-VP6-R	GGTCACATCCTCTCACT	
PoRVA-VP7	PoRVA-VP7-F	GGCTTTAAAAGAGAGAATTTCCG	1002
	PoRVA-VP7-R	GGTCACATCATACAATTCTAATCTAAG	

**Table 2:** The detection results of PoRV in the clinical samples.

Pathogen	Positivity Rate (%)
PoRVA	16.92 (900/5320)
PoRVB	0.51 (27/5320)
PoRVC	12.71 (676/5320)
PoRVH	6.22 (331/5320)
PoRVA + PoRVB	0.06 (3/5320)
PoRVA + PoRVC	3.82 (203/5320)
PoRVA + PoRVH	1.65 (88/5320)
PoRVB + PoRVC	0.04 (2/5320)
PoRVB + PoRVH	0.02 (1/5320)
PoRVC + PoRVH	0.66 (35/5320)
PoRVA + PoRVB + PoRVC	0.02 (1/5320)
PoRVA + PoRVC + PoRVH	0.56 (30/5320)
PoRVA + PoRVB + PoRVC + PoRVH	0.02 (1/5320)

**Table 3:** The homology of the PoRVA VP4, VP6, and VP7 gene sequences.

Gene	Identity Among the Obtained Strains in This Study		Identity Among the Obtained Strains and the Reference Strains	
	Nucleotide (%)	Amino Acid (%)	Nucleotide (%)	Amino Acid (%)
VP4	67.9–99.9	72.1–100.0	60.8–98.7	63.0–99.6
VP6	81.2–99.9	83.3–100.0	73.1–95.9	80.8–98.5
VP7	73.2–100.0	76.5–100.0	70.4–95.5	72.3–97.9

## Data Availability

The data presented in this study are available on request from the corresponding authors due to privacy and ethical reasons.

## Supplementary Materials

The following supporting information can be downloaded at: https://www.mdpi.com/article/10.3390/ani16152292/s1, Table S1: The information on the PoRVA reference strains used in this study; Table S2: The information on the PoRVA strains obtained in this study; Table S3: The information on the PoRVA reference strains used for Maximum Clade Credibility (MCC) tree; Table S4: Comparison of the positivity rates of PoRV in different regions in Guangxi Province using chi-square analysis; Table S5: Comparison of the positivity rates of PoRV in different sample types in Guangxi Province using chi-square analysis.
