# Supplementary material for: Genetic Diversity and Evolution of Porcine Rotavirus Species A in Guangxi Province, Southern China, Between 2022 and 2025
Source: Animals (Basel). 2026 Jul 23;16(15):2292. doi: 10.3390/ani16152292 (PMC13463506; doi:10.3390/ani16152292)
Supplement: Supplementary file 1 [file animals-16-02292-s001.zip › animals-4396313-supplementary.pdf]

Supplementary Materials

Table S1. The information on the PoRVA reference strains used in this study.

No.	Virus strain	Date	Origin	Accession Number	Gene
1	Human-RVA-USA-KC174977-2004	2004	USA	KC174977	VP4
2	Pigeon-RVA-CHN-MG518480-2016	2016	Hainan, CHN	MG518480	VP4
3	Pigeon-RVA-USA-OK185347-2020	2020	USA	OK185347	VP4
4	Human-RVA-UGA-KX655465-2013	2013	UGA	KX655465	VP4
5	Human-RVA-BEL-DQ005111-2003	2003	BEL	DQ005111	VP4
6	Human-RVA-USA-MT796881-1974	1974	USA	MT796881	VP4
7	Human-RVA-RUS-JQ289106-2010	2010	RUS	JQ289106	VP4
8	Human-RVA-BEL-DQ146663-2003	2003	BEL	DQ146663	VP4
9	Human-RVA-ZAF-KJ752472-2007	2007	ZAF	KJ752472	VP4
10	Human-RVA-CHN-KF726067-2013	2013	Hubei, CHN	KF726067	VP4
11	PoRVA-CHN-OQ799682-2022	2022	Shandong, CHN	OQ799682	VP4
12	PoRVA-CHN-OQ799669-2022	2022	Shandong, CHN	OQ799669	VP4
13	PoRVA-CHN-OQ799683-2022	2022	Shandong, CHN	OQ799683	VP4
14	PoRVA-CHN-OQ799705-2022	2022	Guizhou, CHN	OQ799705	VP4
15	Human-RVA-ITA-HQ661115-1997	1997	ITA	HQ661115	VP4
16	Cannie-RVA-JPN-LC326507-1982	1982	JPN	LC326507	VP4
17	PoRVA-THA-HQ268851-2008	2008	THA	HQ268851	VP4
18	PoRVA-CHN-OQ799663-2022	2022	Shandong, CHN	OQ799663	VP4
19	Human-RVA-VNM-LC765808-2017	2017	VNM	LC765808	VP4
20	PoRVA-ITA-KC610703-2009	2009	ITA	KC610703	VP4
21	Human-RVA-THA-LC190489-2014	2014	THA	LC190489	VP4
22	PoRVA-ESP-MH238275-2017	2017	ESP	MH238275	VP4
23	PoRVA-CHN-OQ799706-2022	2022	Shanxi, CHN	OQ799706	VP4
24	PoRVA-CHN-OQ799719-2022	2022	Liaoning, CHN	OQ799719	VP4
25	PoRVA-CHN-OQ799659-2022	2022	Guangdong, CHN	OQ799659	VP4
26	PoRVA-CHN-MK026438-2017	2017	Shanxi, CHN	MK026438	VP4
27	PoRVA-CHN-OQ799698-2022	2022	Liaoning, CHN	OQ799698	VP4
28	PoRVA-CHN-OQ799710-2022	2022	Henan, CHN	OQ799710	VP4
29	PoRVA-CHN-OQ799671-2022	2022	Henan, CHN	OQ799671	VP4
30	Murine-RVA-USA-KJ477174-1982	1982	USA	KJ477174	VP4
31	PoRVA-KOR-MF940546-2006	2006	KOR	MF940546	VP4
32	Bovine-RVA-KOR-HM988969-2010	2010	KOR	HM988969	VP4
33	PoRVA-ESP-KJ450845-2010	2010	ESP	KJ450845	VP4
34	PoRVA-CHN-AY523636-2004	2004	Heilongjiang, CHN	AY523636	VP4
35	PoRVA-CHN-OQ799658-2022	2022	Guangxi, CHN	OQ799658	VP4
36	PoRVA-CHN-OQ799737-2022	2022	Shandong, CHN	OQ799737	VP4
37	PoRVA-CHN-OQ799670-2022	2022	Shandong, CHN	OQ799670	VP4
38	PoRVA-GBR-MN102368-2016	2016	GBR	MN102368	VP4

39	PoRVA-CHN-OQ799743-2022	2022	Gansu, CHN	OQ799743	VP4
40	PoRVA-CHN-OQ799707-2022	2022	Shanxi, CHN	OQ799707	VP4
41	PoRVA-CHN-OQ799656-2022	2022	Guangxi, CHN	OQ799656	VP4
42	PoRVA-CHN-OQ799688-2022	2022	Guangxi, CHN	OQ799688	VP4
43	PoRVA-CHN-OQ799668-2022	2022	Guangxi, CHN	OQ799668	VP4
44	PoRVA-CHN-OQ799667-2022	2022	Guangxi, CHN	OQ799667	VP4
45	PoRVA-AUS-L07886-1993	1993	AUS	L07886	VP4
46	PoRVA-CHN-OQ799733-2022	2022	Liaoning, CHN	OQ799733	VP4
47	PoRVA-CHN-OQ799727-2022	2022	Jiangxi, CHN	OQ799727	VP4
48	PoRVA-JPN-AB573876-2010	2010	JPN	AB573876	VP4
49	PoRVA-CHN-OQ799715-2022	2022	Guizhou, CHN	OQ799715	VP4
50	PoRVA-CHN-OQ799662-2022	2022	Shanxi, CHN	OQ799662	VP4
51	PoRVA-THA-KT727244-2012	2012	THA	KT727244	VP4
52	PoRVA-CHN-OQ799736-2022	2022	Henan, CHN	OQ799736	VP4
53	PoRVA-CHN-OQ799689-2022	2022	Liaoning, CHN	OQ799689	VP4
54	PoRVA-CHN-OQ799745-2022	2022	Liaoning, CHN	OQ799745	VP4
55	PoRVA-CHN-OQ799690-2022	2022	Liaoning, CHN	OQ799690	VP4
56	PoRVA-CHN-OQ799681-2022	2022	Guizhou, CHN	OQ799681	VP4
57	PoRVA-CHN-OQ799697-2022	2022	Guizhou, CHN	OQ799697	VP4
58	PoRVA-CHN-OQ799696-2022	2022	Guizhou, CHN	OQ799696	VP4
1	Cow-RVA-THA-LC133573-1989	1989	THA	LC133573	VP6
2	Human-JPN-JPN-LC328932-2010	2010	JPN	LC328932	VP6
3	Human-RVA-JPN-LC169966-2014	2014	JPN	LC169966	VP6
4	Human-RVA-BRA-KU360936-2010	2010	BRA	KU360936	VP6
5	Human-RVA-ITA-KR632622-2012	2012	ITA	KR632622	VP6
6	Bat-RVA-KEN-NC040411-2015	2015	KEN	NC040411	VP6
7	Llama-RVA-BRA-KY972033-2015	2014	BRA	KY972033	VP6
8	Human-RVA-BRA-MK272921-2003	2003	BRA	MK272921	VP6
9	Human-RVA-BEL-HQ661116-1997	1997	BEL	HQ661116	VP6
10	PoRVA-CHN-OQ799746-2022	2022	Guangxi, CHN	OQ799746	VP6
11	PoRVA-CHN-OQ799788-2022	2022	Guangxi, CHN	OQ799788	VP6
12	PoRVA-CHN-OQ799763-2022	2022	Guangxi, CHN	OQ799763	VP6
13	PoRVA-CHN-OQ799762-2022	2022	Guangxi, CHN	OQ799762	VP6
14	Human-RVA-RUS-JX867347-2010	2010	RUS	JX867347	VP6
15	Human-RVA-THA-LC086762-2013	2013	THA	LC086762	VP6
16	Human-RVA-VNM-KX363297-2016	2016	VNM	KX363297	VP6
17	Human-RVA-USA-KU861383-2016	2016	USA	KU861383	VP6
18	PoRVA-CHN-MG029099-2017	2017	Hunan, CHN	MG029099	VP6
19	PoRVA-CHN-MG066585-2017	2017	Hunan, CHN	MG066585	VP6
20	PoRVA-CHN-OQ799779-2022	2022	Guizhou, CHN	OQ799779	VP6
21	PoRVA-CHN-OQ799771-2022	2022	Guizhou, CHN	OQ799771	VP6
22	PoRVA-CHN-OQ799781-2022	2022	Shandong, CHN	OQ799781	VP6
23	PoRVA-CHN-OQ799879-2022	2022	Liaoning, CHN	OQ799879	VP6
24	PoRVA-CHN-OQ799878-2022	2022	Liaoning, CHN	OQ799878	VP6

25	PoRVA-CHN-OQ799841-2022	2022	Liaoning, CHN	OQ799841	VP6
26	PoRVA-CHN-OQ799874-2022	2022	Liaoning, CHN	OQ799874	VP6
27	PoRVA-CHN-OQ799782-2022	2022	Shandong, CHN	OQ799782	VP6
28	PoRVA-CHN-OQ799866-2022	2022	Shandong, CHN	OQ799866	VP6
29	PoRVA-CHN-OQ799870-2022	2022	Shandong, CHN	OQ799870	VP6
30	PoRVA-CHN-KT820774-2015	2015	Shandong, CHN	KT820774	VP6
31	PoRVA-GBR-MN102370-2019	2019	GBR	MN102370	VP6
32	PoRVA-CHN-KT820766-2015	2015	Shandong, CHN	KT820766	VP6
33	PoRVA-CHN-OQ799774-2022	2022	Gansu, CHN	OQ799774	VP6
34	PoRVA-CHN-OQ799862-2022	2022	Guizhou, CHN	OQ799862	VP6
35	PoRVA-CHN-OQ799834-2022	2022	Henan, CHN	OQ799834	VP6
36	PoRVA-CHN-OQ799751-2022	2022	Guangxi, CHN	OQ799751	VP6
37	PoRVA-CHN-MH021179-2015	2015	Henan, CHN	MH021179	VP6
38	PoRVA-CHN-OQ799753-2022	2022	Guangdong, CHN	OQ799753	VP6
39	PoRVA-CHN-OQ799748-2022	2022	Guangdong, CHN	OQ799748	VP6
40	PoRVA-THA-KU363133-2015	2015	THA	KU363133	VP6
41	PoRVA-THA-EU372798-2008	2008	THA	EU372798	VP6
42	PoRVA-CHN-OQ799853-2022	2022	Tianjin, CHN	OQ799853	VP6
43	PoRVA-CHN-OQ799796-2022	2022	Liaoning, CHN	OQ799796	VP6
44	PoRVA-CHN-OQ799797-2022	2022	Liaoning, CHN	OQ799797	VP6
45	PoRVA-TZA-ON058283-2022	2022	TZA	ON058283	VP6
46	PoRVA-CHN-OQ799867-2022	2022	Liaoning, CHN	OQ799867	VP6
47	PoRVA-CHN-KT820772-2015	2015	Shandong, CHN	KT820772	VP6
48	PoRVA-CHN-OQ799795-2022	2022	Liaoning, CHN	OQ799795	VP6
49	PoRVA-CHN-OQ799790-2022	2022	Liaoning, CHN	OQ799790	VP6
50	PoRVA-CHN-OQ799789-2022	2022	Liaoning, CHN	OQ799789	VP6
1	PoRVA-IND-DQ204743-2006	2006	IND	DQ204743	VP7
2	PoRVA-UGA-KY055421-2017	2017	UGA	KY055421	VP7
3	PoRVA-CHN-OQ743850-2022	2022	Guangxi, CHN	OQ743850	VP7
4	PoRVA-CHN-OQ743881-2022	2022	Guangxi, CHN	OQ743881	VP7
5	PoRVA-CHN-MF462326-2017	2017	Liaoning, CHN	MF462326	VP7
6	PoRVA-CHN-JX498967-2012	2012	Heilongjiang, CHN	JX498967	VP7
7	PoRVA-CHN-OQ743885-2022	2022	Liaoning, CHN	OQ743885	VP7
8	PoRVA-CHN-OQ743919-2022	2022	Hunan, CHN	OQ743919	VP7
9	PoRVA-CHN-OQ743904-2022	2022	Shanxi, CHN	OQ743904	VP7
10	PoRVA-CHN-HM800948-2010	2010	Gansu, CHN	HM800948	VP7
11	PoRVA-IND-KF170900-2013	2013	IND	KF170900	VP7
12	PoRVA-IND-DQ003292-2007	2007	IND	DQ003292	VP7
13	PoRVA-ARG-EF474079-2007	2007	ARG	EF474079	VP7
14	PoRVA-USA-AB180972-2016	2016	USA	AB180972	VP7
15	PoRVA-CHN-OQ743863-2022	2022	Shandong, CHN	OQ743863	VP7
16	PoRVA-CHN-OQ743873-2022	2022	Shandong, CHN	OQ743873	VP7
17	PoRVA-USA-X06386-1993	1993	USA	X06386	VP7
18	PoRVA-CHN-JX498956-2012	2012	Heilongjiang, CHN	JX498956	VP7

19	Human-RVA-THA-KT007665-2015	2015	THA	KT007665	VP7
20	Human-RVA-JPN-LC095910-2019	2019	JPN	LC095910	VP7
21	PoRVA-CHN-OQ743859-2022	2022	Guangxi, CHN	OQ743859	VP7
22	PoRVA-SLO-DQ629928-2016	2016	SLO	DQ629928	VP7
23	PoRVA-JPN-LC081150-2016	2016	JPN	LC081150	VP7
24	Human-RVA-AUS-JX027895-2014	2014	AUS	JX027895	VP7
25	Human-RVA-TUR-KU258001-2016	2016	TUR	KU258001	VP7
26	Human-RVA-IND-MH560416-2019	2019	IND	MH560416	VP7
27	PoRVA-CHN-OQ743949-2022	2022	Zhejiang, CHN	OQ743949	VP7
28	PoRVA-CHN-OQ743908-2022	2022	Zhejiang, CHN	OQ743908	VP7
29	PoRVA-CHN-OQ743857-2022	2022	Shandong, CHN	OQ743857	VP7
30	Human-RVA-BRA-MG407650-2015	2015	BRA	MG407650	VP7
31	PoRVA-IND-KT277524-2015	2015	IND	KT277524	VP7
32	PoRVA-BRA-KT310239-2016	2016	BRA	KT310239	VP7
33	PoRVA-CHN-OQ743991-2022	2022	Jiangxi, CHN	OQ743991	VP7
34	PoRVA-CHN-OQ743954-2022	2022	Guangdong, CHN	OQ743954	VP7
35	PoRVA-CHN-OQ743952-2022	2022	Tianjin, CHN	OQ743952	VP7
36	PoRVA-MEX-M23194-1994	1994	MEX	M23194	VP7
37	PoRVA-CHN-OQ743990-2022	2022	Liaoning, CHN	OQ743990	VP7
38	PoRVA-CHN-OQ743989-2022	2022	Liaoning, CHN	OQ743989	VP7
39	PoRVA-ITA-DQ062572-2006	2006	ITA	DQ062572	VP7
40	PoRVA-KOR-JX971568-2012	2012	KOR	JX971568	VP7
41	PoRVA-CHN-AY538665-2004	2004	Heilongjiang, CHN	AY538665	VP7
42	PoRVA-USA-X04613-2003	2003	USA	X04613	VP7
43	PoRVA-CHN-OQ743981-2022	2022	Liaoning, CHN	OQ743981	VP7
44	PoRVA-CHN-OQ743847-2022	2022	Guangxi, CHN	OQ743847	VP7
45	PoRVA-CHN-OQ743970-2022	2022	Liaoning, CHN	OQ743970	VP7
46	PoRVA-CHN-KT820775-2016	2016	Shandong, CHN	KT820775	VP7
47	PoRVA-CHN-OQ743958-2022	2022	Zhejiang, CHN	OQ743958	VP7
48	PoRVA-ITA-AY766085-2005	2005	ITA	AY766085	VP7
49	PoRVA-CHN-OQ743849-2022	2022	Guangxi, CHN	OQ743849	VP7
50	PoRVA-CHN-OQ743948-2022	2022	Liaoning, CHN	OQ743948	VP7
51	PoRVA-VNM-JF781163-2017	2017	VNM	JF781163	VP7
52	Human-RVA-RUS-KF648937-2012	2012	RUS	KF648937	VP7
53	Human-RVA-JPN-LC477373-2018	2018	JPN	LC477373	VP7
54	Human-RVA-USA-KC782524-2010	2010	USA	KC782524	VP7
55	PoRVA-CHN-KC113248-2013	2013	Hubei, CHN	KC113248	VP7
56	PoRVA-CHN-KY649279-2018	2018	Henan, CHN	KY649279	VP7
57	PoRVA-CHN-OQ743937-2022	2022	Guizhou, CHN	OQ743937	VP7
58	PoRVA-CHN-OQ743853-2022	2022	Liaoning, CHN	OQ743853	VP7
59	PoRVA-CHN-KJ126835-2014	2014	Hebei, CHN	KJ126835	VP7
60	PoRVA-CHN-OQ743941-2022	2022	Shandong, CHN	OQ743941	VP7
61	PoRVA-CHN-OQ743871-2022	2022	Liaoning, CHN	OQ743871	VP7
62	PoRVA-CHN-OQ743877-2022	2022	Guangxi, CHN	OQ743877	VP7

Note: ARG: Argentina; AUS: Australia; BEL: Belgium; BRA: Brazil; CHN: China; CZE: Czech; ESP: Spain; GBR: Britain; IND: India; ITA: Italy; JPN: Japan; KEN: Kenya; KOR: Korea; RUS: Russia; MEX: Mexico; THA: Thailand; UGA: Uganda; USA: The United States of America; VNM: Vietnam; SLO: Slovenia; ZAF: South Africa. The same as below.

Table S2. The information on the PoRVA strains obtained in this study.

No.	Virus strain	Date	Origin	Accession Number			Genotype
				VP4	VP6	VP7	(VP7/VP4VP6)
1	PoRVA/GXBS1/2022/China	2022	Guuangxi, CHN	PV655424	PV655191	PV655243	G9P[13]I5
2	PoRVA/GXBS2/2022/China	2022	Guuangxi, CHN	PV655425	PV655192	PV655244	G9P[13]I5
3	PoRVA/GXBS3/2022/China	2022	Guuangxi, CHN	PV655426	PV655193	PV655245	G9P[23]I5
4	PoRVA/GXGG1/2022/China	2022	Guuangxi, CHN	PV655427	PV655194	PV655246	G2P[23]I5
5	PoRVA/GXGG2/2022/China	2022	Guuangxi, CHN	PV655428	PV655195	PV655247	G5P[23]I5
6	PoRVA/GXGG3/2022/China	2022	Guuangxi, CHN	PV655429	PV655196	PV655248	G9P[13]I5
7	PoRVA/GXNN1/2022/China	2022	Guuangxi, CHN	PV655430	PV655197	PV655249	G5P[13]I5
8	PoRVA/GXBS4/2022/China	2022	Guuangxi, CHN	PV655431	PV655198	PV655250	G3P[6]I5
9	PoRVA/GXBS5/2022/China	2022	Guuangxi, CHN	PV655432	PV655199	PV655251	G9P[13]I5
10	PoRVA/GXNN2/2022/China	2022	Guuangxi, CHN	PV655433	PV655200	PV655252	G5P[23]I5
11	PoRVA/GXBS6/2022/China	2022	Guuangxi, CHN	PV655434	PV655201	PV655253	G9P[13]I5
12	PoRVA/GXBS7/2022/China	2022	Guuangxi, CHN	PV655435	PV655202	PV655254	G11P[13]I5
13	PoRVA/GXCZ1/2023/China	2023	Guuangxi, CHN	PV655436	PV655203	PV655255	G9P[23]I5
14	PoRVA/GXQZ1/2023/China	2023	Guuangxi, CHN	PV655437	PV655204	PV655256	G26P[13]I5
15	PoRVA/GXGG1/2023/China	2023	Guuangxi, CHN	PV655438	PV655205	PV655257	G9P[13]I5
16	PoRVA/GXGG2/2023/China	2023	Guuangxi, CHN	PV655439	PV655206	PV655258	G9P[13]I5
17	PoRVA/GXBS1/2023/China	2023	Guuangxi, CHN	PV655440	PV655207	PV655259	G4P[6]I1
18	PoRVA/GXBS2/2023/China	2023	Guuangxi, CHN	PV655441	PV655208	PV655260	G4P[6]I1
19	PoRVA/GXBS3/2023/China	2023	Guuangxi, CHN	PV655442	PV655209	PV655261	G4P[6]I1
20	PoRVA/GXLZ1/2023/China	2023	Guuangxi, CHN	PV655443	PV655210	PV655262	G12P7I5
21	PoRVA/GXWZ1/2023/China	2023	Guuangxi, CHN	PV655444	PV655211	PV655263	G5P[13]I5
22	PoRVA/GXBS4/2023/China	2023	Guuangxi, CHN	PV655445	PV655212	PV655264	G5P[13]I5
23	PoRVA/GXNN1/2023/China	2023	Guuangxi, CHN	PV655446	PV655213	PV655265	G9P[23]I5
24	PoRVA/GXNN2/2023/China	2023	Guuangxi, CHN	PV655447	PV655214	PV655266	G9P[13]I5
25	PoRVA/GXNN3/2023/China	2023	Guuangxi, CHN	PV655448	PV655215	PV655267	G9P[23]I5
26	PoRVA/GXNN4/2023/China	2023	Guuangxi, CHN	PV655449	PV655216	PV655268	G9P[13]I5
27	PoRVA/GXBS5/2023/China	2023	Guuangxi, CHN	PV655450	PV655217	PV655269	G11P[13]I5
28	PoRVA/GXYL1/2023/China	2023	Guuangxi, CHN	PV655451	PV655218	PV655270	G9P[13]I5
29	PoRVA/GXBS6/2023/China	2023	Guuangxi, CHN	PV655452	PV655219	PV655271	G4P[6]I1
30	PoRVA/GXBS7/2023/China	2023	Guuangxi, CHN	PV655453	PV655220	PV655272	G4P[13]I5
31	PoRVA/GXCZ2/2023/China	2023	Guuangxi, CHN	PV655454	PV655221	PV655273	G26P[6]I1
32	PoRVA/GXBS8/2023/China	2023	Guuangxi, CHN	PV655455	PV655222	PV655274	G5P[23]I5
33	PoRVA/GXCZ3/2023/China	2023	Guuangxi, CHN	PV655456	PV655223	PV655275	G4P[13]I5
34	PoRVA/GXBS9/2023/China	2023	Guuangxi, CHN	PV655457	PV655224	PV655276	G3P[13]I5
35	PoRVA/GXHZ1/2024/China	2024	Guuangxi, CHN	PV655458	PV655225	PV655277	G9P[13]I5
36	PoRVA/GXFCG1/2024/China	2024	Guuangxi, CHN	PV655459	PV655226	PV655278	G5P[13]I5
37	PoRVA/GXBS1/2024/China	2024	Guuangxi, CHN	PV655460	PV655227	PV655279	G9P[23]I5
38	PoRVA/GXNN1/2024/China	2024	Guuangxi, CHN	PV655461	PV655228	PV655280	G9P[13]I5
39	PoRVA/GXNN2/2024/China	2024	Guuangxi, CHN	PV655462	PV655229	PV655281	G5P[6]I5

40	PoRVA/GXYL1/2024/China	2024	Guuangxi, CHN	PV655463	PV655230	PV655282	G11P[13]I5
41	PoRVA/GXYL2/2024/China	2024	Guuangxi, CHN	PV655464	PV655231	PV655283	G9P[13]I5
42	PoRVA/GXGG1/2024/China	2024	Guuangxi, CHN	PV655465	PV655232	PV655284	G4P[13]I5
43	PoRVA/GXGG2/2024/China	2024	Guuangxi, CHN	PV655466	PV655233	PV655285	G4P[13]I5
44	PoRVA/GXYL3/2024/China	2024	Guuangxi, CHN	PV655467	PV655234	PV655286	G5P[13]I5
45	PoRVA/GXYL4/2024/China	2024	Guuangxi, CHN	PV655468	PV655235	PV655287	G9P[23]I5
46	PoRVA/GXGL1/2024/China	2024	Guuangxi, CHN	PV655469	PV655236	PV655288	G5P[13]I5
47	PoRVA/GXBH1/2025/China	2025	Guuangxi, CHN	PV655470	PV655237	PV655289	G5P[13]I5
48	PoRVA/GXLZ1/2025/China	2025	Guuangxi, CHN	PV655471	PV655238	PV655290	G5P[13]I5
49	PoRVA/GXCZ1/2025/China	2025	Guuangxi, CHN	PV655472	PV655239	PV655291	G9P[13]I5
50	PoRVA/GXHZ1/2025/China	2025	Guuangxi, CHN	PV655473	PV655240	PV655292	G9P[13]I5
51	PoRVA/GXHC1/2025/China	2025	Guuangxi, CHN	PV655474	PV655241	PV655293	G9P[13]I5
52	PoRVA/GXLB1/2025/China	2025	Guuangxi, CHN	PV655475	PV655242	PV655294	G9P[13]I5

Table S3. The information on the PoRVA reference strains used for Maximum Clade Credibility (MCC) tree.

No.	Virus strain	Date	Origin	Accession Number	Genotype
1	Human-RVA-USA-KC174977-2004	2004	USA	KC174977	P[11]
2	Pigeon-RVA-CHN-MG518480-2016	2016	Hainan, CHN	MG518480	P17
3	Pigeon-RVA-USA-OK185347-2020	2020	USA	OK185347	P17
4	Human-RVA-UGA-KX655465-2013	2013	UGA	KX655465	P[4]
5	Human-RVA-BEL-DQ005111-2003	2003	BEL	DQ005111	P[8]
6	Human-RVA-USA-MT796881-1974	1974	USA	MT796881	P[8]
7	Human-RVA-RUS-JQ289106-2010	2010	RUS	JQ289106	P[6]
8	Human-RVA-BEL-DQ146663-2003	2003	BEL	DQ146663	P[6]
9	Human-RVA-ZAF-KJ752472-2007	2007	ZAF	KJ752472	P[6]
10	Human-RVA-CHN-KF726067-2013	2013	Hubei, CHN	KF726067	P[6]
11	PoRVA-CHN-OQ799682-2022	2022	Shandong, CHN	OQ799682	P[6]
12	PoRVA-CHN-OQ799705-2022	2022	Guizhou, CHN	OQ799705	P[3]
13	Human-RVA-ITA-HQ661115-1997	1997	ITA	HQ661115	P[3]
14	Cannie-RVA-JPN-LC326507-1982	1982	JPN	LC326507	P[3]
15	PoRVA-THA-HQ268851-2008	2008	THA	HQ268851	P[23]
16	PoRVA-CHN-OQ799663-2022	2022	Shandong, CHN	OQ799663	P[23]
17	Human-RVA-VNM-LC765808-2017	2017	VNM	LC765808	P[23]
18	PoRVA-ITA-KC610703-2009	2009	ITA	KC610703	P[23]
19	Human-RVA-THA-LC190489-2014	2014	THA	LC190489	P[23]
20	PoRVA-ESP-MH238275-2017	2017	ESP	MH238275	P[23]
21	PoRVA-CHN-OQ799719-2022	2022	Liaoning, CHN	OQ799719	P[23]
22	PoRVA-CHN-OQ799671-2022	2022	Henan, CHN	OQ799671	P[23]
23	Murine-RVA-USA-KJ477174-1982	1982	USA	KJ477174	P[16]
24	PoRVA-KOR-MF940546-2006	2006	KOR	MF940546	P[7]
25	Bovine-RVA-KOR-HM988969-2010	2010	KOR	HM988969	P[7]
26	PoRVA-ESP-KJ450845-2010	2010	ESP	KJ450845	P[7]
27	PoRVA-CHN-OQ799670-2022	2022	Shandong, CHN	OQ799670	P[7]
28	PoRVA-GBR-MN102368-2016	2016	GBR	MN102368	P[7]
29	PoRVA-CHN-OQ799743-2022	2022	Gansu, CHN	OQ799743	P[7]
30	PoRVA-CHN-OQ799667-2022	2022	Guangxi, CHN	OQ799667	P[7]
31	PoRVA-AUS-L07886-1993	1993	AUS	L07886	P[13]
32	PoRVA-CHN-OQ799727-2022	2022	Jiangxi CHN	OQ799727	P[13]
33	PoRVA-JPN-AB573876-2010	2010	JPN	AB573876	P[13]
34	PoRVA-CHN-OQ799662-2022	2022	Shanxi, CHN	OQ799662	P[13]
35	PoRVA-THA-KT727244-2012	2012	THA	KT727244	P[13]
36	PoRVA-CHN-OQ799736-2022	2022	Henan, CHN	OQ799736	P[13]

Table S4. Comparison of the positivity rates of PoRV in different regions in Guangxi Province using chi-square analysis.

Region	Number	Positive rate % (number of positive sample)			
		PoRVA	PoRVB	PoRVC	PoRVH
Liuzhou	506	20.95 (106)^{a}	1.19 (6)^{a}	16.60 (84)^{c}	19.76 (100)^{a}
Guilin	226	15.93 (36)^{b}	0 (0)	0 (0)	0 (0)
Chongzuo	383	16.19 (62)^{b}	0 (0)	8.62 (33)^{d}	2.61 (10)^{d}
Beihai	234	4.27 (10)^{d}	0 (0)	0 (0)	0 (0)
Fangchenggang	102	10.78 (11)^{c}	0 (0)	3.92 (4)^{de}	0 (0)
Hezhou	113	6.19 (7)^{cd}	0 (0)	10.62 (12)^{cd}	15.04(17)^{ab}
Laibin	223	8.97 (20)^{c}	0 (0)	0 (0)	0 (0)
Guigang	476	21.85 (104)^{a}	0 (0)	21.85 (104)^{ab}	5.88 (28)^{c}
Baise	1,202	22.05 (265)^{a}	1.08 (13)^{a}	20.55 (247)^{b}	5.57 (67)^{c}
Hechi	253	13.83 (35)^{bc}	0 (0)	1.58 (4)^{e}	0 (0)
Yulin	419	13.13 (55)^{bc}	0.48 (2)^{a}	6.92 (29)^{d}	10.74 (45)^{b}
Qinzhou	304	13.49 (41)^{bc}	0.33 (1)^{a}	0 (0)	10.86 (33)^{b}
Wuzhou	236	4.24 (10)^{d}	0 (0)	0 (0)	5.08 (12)^{c}
**Total**	**5,320**	**16.92 (900)^{A}**	**0.51 (27)^{D}**	**12.71 (676)^{B}**	**6.22 (331)^{C}**

Note: In the same column for different PoRV species, values with different letter superscripts mean significant difference ($p \leqslant 0.05$); While with the same or no letter superscripts mean no significant difference ($p>0.05$). In the last row, values with different letter superscripts mean significant difference ($p \leqslant 0.05$). The same as Table S5.

Table S5. Comparison of the positivity rates of PoRV in different sample types in Guangxi Province using chi-square analysis.

Sample	Number	Positive rate % (number of positive sample)			
		PoRVA	PoRVB	PoRVC	PoRVH
Fecal swab	4,523	17.31 (783)^{a}	0.51 (23)^{a}	13.15 (595)^{a}	6.28 (284)^{a}
Tissue	817	14.32 (117)^{b}	0.49 (4)^{a}	9.91 (81)^{b}	6.75 (47)^{a}
**Total**	**5,320**	**16.92 (900)^{A}**	**0.51 (27)^{D}**	**12.71 (676)^{B}**	**6.22 (331)^{C}**
